# Obstetric Outcome of Induction of Labour in a Tertiary Hospital in Nigeria: A Five-Year Retrospective Cross-Sectional Study

**DOI:** 10.23958/ijirms/vol08-i07/1706

**Published:** 2023-07-06

**Authors:** Harrison Chiro Ugwuoroko, George Uchenna Eleje, Chigozie Geoffrey Okafor, Zebulon Chiawolamoke Okechukwu, Ahizechukwu Chigoziem Eke, Chukwuemeka Chukwubuikem Okoro, Lazarus Ugochukwu Okafor, Chidinma Charity Okafor, Chukwudi Anthony Ogabido, Tobechi Kingsley Njoku, Chukwudubem Chinagorom Onyejiaka, Adanna Vivian Egwim, Hillary Ikechukwu Obiagwu, JohnBosco Emmanuel Mamah, Chinedu Lawrence Olisa, Nnanyerugo Livinus Onah, Gerald Okanandu Udigwe

**Affiliations:** 1 Department of Obstetrics and Gynaecology, Nnamdi Azikiwe University Teaching Hospital, Nnewi, Anambra State, Nigeria.; 2 Department of Obstetrics and Gynaecology, Nnamdi Azikiwe University Awka, Anambra State, Nigeria.; 3 Division of Maternal and Fetal Medicine, Department of Gynaecology and Obstetrics, Johns Hopkins University School of Medicine, Baltimore, USA.; 4 Department of Psychiatry, Leicestershire Partnership NHS Trust, Leicester, United Kingdom.; 5 Department of Obstetrics and Gynaecology, Alex Ekwueme Federal University Teaching Hospital, Abakiliki, Nigeria.; 6 Department of Obstetrics and Gynaecology, Enugu State Teaching Hospital Parklane, Enugu State, Nigeria.

## Abstract

**Background:**

Induction of labour has remained one of the most valuable interventions in obstetric practice. Over the years, the proportion of women undergoing induction of labour (IOL) has been on a steady increase. The significance to obstetrics practice as well as its maternal and perinatal outcomes are sacrosanct, hence the need for its periodic review.

**Objective:**

To determine the obstetric outcomes of induction of labour.

**Methods:**

A five-year retrospective study of all cases of induction of labour at the maternity unit of Nnamdi Azikiwe University Teaching Hospital (NAUTH), Nnewi, Nigeria between January 1st 2017 and 31st December 2021. The labour ward’s records were assessed to determine the total number of women who had induction of labour during the study period. Women whose case files could be not retrieved were excluded. The folder numbers of the patients were extracted and their case files retrieved from the medical records department of the hospital. The primary outcomes measures were the indications and the methods of induction of labour, while the secondary outcome measures were the mode of delivery, cause of failed induction, and the perinatal outcome. Data were obtained using proformas and analysed using statistical packages for social sciences (SPSS) version 26.0 IBM corporation.

**Result:**

A total of 3,638 deliveries were taken during the period under review and 168 patients had induction of labour giving an overall prevalence of 4.6% (46/1000 deliveries). Induction of labour was successful in 71.2% of cases. Misoprostol was used in 90.4% of cases as an induction agent. The commonest indication for induction of labour was postdate pregnancy (53.8%). Failed induction was due to fetal distress, poor progress of labour from cephalopelvic disproportion/malposition and failed cervical ripening. In about 72% of deliveries, there was good perinatal outcome, 10.3% of babies had moderate to severe asphyxia while 1.3% had neonatal death.

**Conclusion:**

Induction of labour is a safe and beneficial procedure in obstetrics. However, it can be associated with adverse obstetric outcomes.

## Introduction

Induction of labour is one of the most important interventions in obstetric practice ^[1–3]^. It is defined as the initiation of uterine contractions after the age of viability but before the onset of natural labour by medical and/or surgical means for the purpose of normal delivery ^[1]^. Induction of labour is planned initiation of labour prior to its spontaneous onset ^[3,4]^. It is usually indicated when the benefits of delivery of the fetus outweighs the risk of continuing the pregnancy ^[3–5]^. It should only be performed if the chance of success is high and if the risks of the process to the mother and/or fetus are minimal ^[4]^.

The rate has been on the increase, it varies within countries and among local hospitals ^[6,5]^. Nearly 20–25% of deliveries in the United Kingdom are preceded by induction of labour (IOL) ^[4]^. In Nigeria, rates of 4.9% and 11.5% have been reported in Jos and Ogoja respectively ^[3,5]^. The possible indications for induction of labour include a range of conditions associated with maternal or fetal compromise ^[7]^. The most common indication for IOL is prolonged pregnancy or postdated pregnancy ^[4]^. Induction for this reason has been shown to reduce the likelihood of perinatal mortality ^[3,4]^. Prolonged pregnancy accounted for 45.8% of IOL in Cross River, Nigeria ^[5]^. Prelabour rupture of membranes (PROM) is another common indication for IOL especially at term ^[4]^. Other indications for IOL include pre- eclampsia and other maternal hypertensive disorders, fetal growth restriction, diabetes mellitus, renal disease, deteriorating maternal illness, intrahepatic cholestasis of pregnancy, and intra uterine fetal death, to name but few ^[1,4,8]^.

Induction when successful results in vaginal delivery. Though, it is not free of adverse outcome, IOL is an efficient and safe method of vaginal delivery ^[9]^. It can sometimes be complicated with potential fetal and maternal risks such as increased rate of abdominal delivery, excessive uterine activity, fetal heart rate abnormalities, uterine rupture, birth asphyxia, intrauterine fetal death (IUFD), maternal water intoxication, preterm delivery due to erroneous estimation of gestational dates, and possible cord prolapse ^[5,7,10]^

This study was aimed to determine the rate, indications and obstetric outcome of induction of labour in NAUTH Nnewi, Nigeria over a 5-year period.

## Materials and Method

### Study design:

This is a retrospective cross-sectional study.

### Study population:

The study was conducted among women that had induction of labour in NAUTH Nnewi, Nigeria between 1st January, 2017 and 31st December 2021.

### Study setting:

This study was conducted in the obstetric/labour ward unit of Nnamdi Azikiwe University Teaching Hospital, Nnewi, Nigeria. NAUTH is a 400 bedded tertiary institution located in Anambra state, South Eastern Nigeria. It provides an excellent emergency obstetrics services on 24-hour bases as well as out-patient obstetrics services, comprehensive and specialized health services to inhabitants of Anambra state and the surrounding states, in Nigeria. The hospital is a training centre for postgraduate and undergraduate studies.

### Inclusion criteria:

This included women who had induction of labour at NAUTH within the study period.

### Exclusion criteria:

Those excluded from the study were participants whose case files could not be retrieved and those with incomplete documentations.

### Sample technique:

Non-random sampling approach was used. All available case files from the medical record department were examined.

#### Study outcome measures:

The indications and the methods of induction of labour, the mode of delivery, cause of failed induction, and the perinatal outcome.

### Study Procedures:

The labour ward, obstetric theatre and lying-in ward records were reviewed to identify patients that had induction of labour during the study period. The patients’ case records were retrieved from the hospital medical record department using the folder number. The patients’ socio-demographic variables, indications for induction of labour, methods of induction of labour and obstetric outcomes, were retrieved from the patients’ case files using proformas.

#### Statistical Analysis

The data was analysed using the statistical package for social sciences (SPSS) computer software version 26.0 IBM corporation. A p -value of <0.05 was considered statistically significant.

## Results

A total of 3,638 deliveries were taken during the period under review and 168 patients had induction of labour giving an overall prevalence of 4.6% (46/1000 deliveries). Table 1 shows the sociodemographic variables of the participants. The modal age range for the selected subjects was 25–29 years. With respect to the age range, 3.8% of them were ≤ 20 years while 1.9% were ≥ 40 years. Also, 46.8% of the subjects had tertiary education while 1.3% had no formal education. This is shown in Table 1.

The obstetric variables of the subjects are shown in Table 2. Majority of the participants 123 (78.8%) were booked and received antenatal care in our facility while 33 (21.2%) were unbooked. Nulliparity accounted for 49.4% of cases, while 2.5% of the subjects were grand multiparous. Most of the subjects (50%) were induced at a gestational age of 41 weeks and above, however, 5.8% were induced between 28 weeks and 32 weeks gestation.

With regard to the indication for induction of labour, 54% of the subjects were induced on account of postdated pregnancy. Other indications were; hypertensive diseases in pregnancy (7.7%), preterm PROM (3.8%), term PROM (5.8%), Intra-uterine fetal death (17.3%) while 11.5% were due to other indications such as diabetes mellitus in pregnancy, and other medical diseases in pregnancy. Vast majority of the participants (90.4%) were induced with misoprostol, 7.7% were induced with intracervical extra amniotic Foley catheter/amniotomy/oxytocin infusion while amniotomy and oxytocin infusion following membrane sweep constituted the least method of induction of labour (1.9%). This is shown in Table 3.

Grand multiparous subjects had 100% success rate, nulliparous had 58.4% while multipara had a mean success rate of 88.9%. Those induced on account of hypertensive diseases had 100% success rate, followed by those with IUFD (88.9%) while the least was seen in preterm PROM (50%). Participants who had membrane sweep/amniotomy /oxytocin infusion had 100% success rate, those who had Foley catheter insertion/amniotomy/oxytocin infusion had a success rate of 66.7%. The use of misoprostol was associated with 70.2% success rate. The success rate was significantly affected by parity and indications for the induction of labour. This is shown in Table 4.

Majority of the women (71.2%) had a successful induction of labour resulting in vaginal delivery while 28.8% had failed induction of labour resulting in emergency caesarean delivery. Poor progress of labour due to cephalopelvic disproportion and malposition constituted the most indication for caesarean delivery (48.8%), while the least indication as a result of failed cervical ripening (17.8%). This is shown in table 5. In terms of neonatal outcome, 72.4% of babies delivered had good perinatal outcome, 10.3% had moderate to severe asphyxia while 1.3% had neonatal death. This is shown in Table 6.

## Discussion

Induction of labour is one of the most useful interventions in modern day obstetrics practice. The rate of induction of labour in this study was 4.6% (46/1000 deliveries). This is closely similar to rates of 4.5% reported by Oyebode et al in Jos, but much lower than rates of 11.5% and 12.7% reported in Ogoja and Ibadan in Nigeria respectively ^[3,5,11]^. Higher rates of 23.7%, 27% and 31.1% have been reported in Canada, the United States and Australia respectively ^[12–14]^. Although these are national based studies, the higher rates observed in these developed countries could be attributable to the increase in elective induction seen in these countries as well as the use of other effective agents such as prostaglandin gels for induction of labour.

Majority (53.8%) of the inductions of labour in our study were done for postdated pregnancies. This was similar to previous studies done at Ogoja, Jos and Maiduguri in Nigeria where it accounted for 45.8%, 44.5% and 46.8% respectively and 53.8% as in this study ^[3,5,15]^. This varied with the findings of Lueth et al in Ethiopia where prolonged rupture of membranes was identified as the leading indication ^[16]^. Other notable indications for induction of labour in this study included intrauterine fetal death, hypertensive diseases and premature rupture of membranes and this is in keeping with findings in previous studies in Jos, Ogoja, Maiduguri (all in Nigeria) and Ethopia ^[3,5,11,17]^.

Misoprostol was the commonest agent for induction of labour in this study. This was also the commonest method reported in similar studies in Ogoja and Ibadan in Nigeria where it was associated with shorter induction delivery interval than the other methods ^[5,11]^. It was used alone or followed by oxytocin titration in those with favourable cervix but without adequate uterine contractions. In all cases where misoprostol was used, 50mcg was used via vaginal or sublingual routes. Although, the World Health Organisation (WHO) recommended 25mcg of misoprostol for induction of labour and studies have shown similar outcomes for vaginal and sublingual routes ^[18]^. However, 25mcg preparation is not readily available in our environment as it is only feasible to get 50mcg from the available 200mcg preparation. The success rate for the use of misoprostol in this study was 70.2% which was similar to 75.9% reported in Ogoja in Nigeria ^[5]^. Membrane sweeping and intracervical extra amniotic Foley catheter were methods used for cervical ripening in our subjects who were not suitable for misoprostol use such as grand multiparous women and those with prior uterine surgeries who were at increased risk of uterine rupture and these were followed by judicious oxytocin use for induction of labour in these subjects.

The overall success rate for induction of labour in this study was 71.2% which was similar to 67.6% reported by Oshodi et al and 65% reported by Yimer et al, but lower than rate of 82.2% reported by Oyebode et al. ^[5,19 20]^ Multiparous women were found to have higher chances of successful vaginal delivery with mean success rate of 91.7% than nulliparous women with rate of 58.4% which was in keeping with the findings of Oyebode et al where nulliparous women had highest rate of operative delivery ^[3]^. With respect to indications, those induced on account of hypertensive diseases had the highest success rate (100%) followed by those who had IUFD (88.9%). This could be explained by the fact that in many cases of IUFD, severe preeclampsia/eclampsia, physiological process of labour might have been initiated prior to induction of labour. More so, those with IUFD were given longer window of time for induction since fetal indications for surgery like fetal distress were eliminated. Oyebode et al reported highest success rates in women with IUFD and PROM ^[3]^.

In this study, 28.8% of the subjects had a failed induction of labour that resulted in operative delivery on account of fetal distress, poor progress of labour from cephalopelvic disproportion/malposition and failed cervical ripening which was similar to findings in Jos and Ogoja, Nigeria ^[3,5]^.

Overall, 72.4% of babies had good perinatal outcome with good Apgar score, 10.3% of babies had moderate to severe asphyxia necessitating admission in special care baby unit, while 2(1.3%) had neonatal deaths. These findings are similar to findings in previous studies ^[3,19]^. Fetal distress from uterine hyperstimulation and cephalopelvic disproportion may have accounted for the birth asphyxia seen in these babies.

A strength of this study was the inclusion of all cases of induction of labour at retrieval of case files during the study periods. The definition of variables were the same across the study periods and all the outcome diagnosis were crosschecked and validated against relevant registers and medical records. Several limitations must be addressed. The main limitation was the low number of complications observed and the short follow-up of the newborns, making the study underpowered to detect small changes over time. The type (brand) of misoprostol used may have varied for the study periods. Further, it cannot be ruled out that morbidity or complications observed during induction of labour may have been applied slightly differently across the study periods. Lastly, the study did not include subsequent pregnancy labour outcome follow-up data.

## Conclusion

Induction of labour is a safe and beneficial procedure in obstetrics. It is employed in high-risk pregnancies when the benefits of early delivery outweigh the risks of continuing the pregnancy. Although, it is a relatively safe procedure, it can be associated with failure and adverse fetal/maternal outcomes. Hence, proper patient selection and adequate monitoring are sine qua non to achieving positive outcome.

## Figures and Tables

**Table 1:** Socio-demographic variables of the studied population

Variable	Frequency	Percentage (%)
**Age (years)**		
<20	6	3.8
20–24	25	16.0
25–29	52	33.3
30–34	46	29.5
35–39	24	15.4
≥40	3	1.9
**Total**	**156**	**100**
**Marital status**		
Single	3	1.9
Married	153	98.1
Divorced	0	0
**Total**	**156**	**100**
**Level of education**		
No formal education	2	1.3
Primary	21	13.5
Secondary	60	38.5
Tertiary	73	46.8
**Total**	**156**	**100**
**Occupation**		
Unemployed	21	13.5
Trading	47	29.7
Civil servant	37	23.7
Others	51	32.7
**Total**	**156**	**100**

**Table 2:** Analysis of some obstetrics variables

Variable	Frequency	Percentage (%)
**Booking status**		
Booked	123	78.8
Un-booked	33	21.2
**Total**	**156**	**100**
**Parity**		
0	77	49.4
1	33	21.2
2	21	13.5
3	9	5.8
4	12	7.7
≥5	4	2.5
**Total**	**156**	**100.0**
**Gestational age at induction of labour(weeks)**		
28–32	9	5.8
33–36	42	26.9
37–40	27	17.3
≥41	78	50.0
**Total**	**156**	**100.0**

**Table 3:** Indications and methods of induction of labour

Variable	Frequency	Percentage (%)
**Main indications**		
Postdate	84	53.8
IUFD	27	17.30
Hypertensive disease	12	7.7
Term PROM	9	5.8
Preterm PROM	6	3.8
Others	18	11.5
**Total**	**156**	**100.0**
**Method of induction of labour**		
Use of misoprostol	141	90.4
Foley catheter/Amniotomy/Oxytocin	12	7.7
Membrane sweep/Amniotomy/Oxytocin	3	1.9
**Total**	**156**	**100.0**

* IUFD- intrauterine foetal death,

* PROM- Prelabour rupture of membrane.

**Table 4:** Outcome of induction of labour according to parity, indications and methods of induction

Variable	Mode of delivery		X^2^	p-value
	Vaginal	Caesarean section		
**Parity**			18.232	0.003
0	45	32		
1	27	6		
2	14	7		
3	9	0		
4	12	0		
≥5	4	0		
**Main indications**			12.506	0.028
Postdated pregnancy	54	30		
IUFD	24	3		
Hypertensive disease	12	0		
Term PROM	6	3		
Preterm PROM	3	3		
Others	12	6		
**Method of induction of labour**			1.346	0.510
Use of misoprostol	99	42		
Foley catheter/Amniotomy/Oxytocin	8	4		
Membrane sweep/Amniotomy/Oxytocin	3	0		

**Table 5:** Mode of delivery and indication for caesarean section/reason for failed induction

Variable	Frequency	Percentage (%)
**Mode of delivery**		
Vaginal	111	71.2
Caesarean section	45	28.8
**Total**	**156**	**100.0**
**Indications for caesarean section**		
Foetal distress	15	33.3
Poor progress	22	48.8
Failed cervical ripening	8	17.8
**Total**	**45**	**100.0**

**Table 6:** Perinatal outcome

Perinatal outcome	Frequency	Percentage (%)
Good outcome/APGAR score	113	72.4
Moderate to severe asphyxia	16	10.3
Neonatal death	2	1.3

## Data Availability

Research data are available upon request from the authors.

## Abbreviations

IOL: Induction of Labour
IUFD: Intrauterine Fetal Death
NAUTH: Nnamdi Azikiwe University Teaching Hospital
PROM: Prelabour Rupture of Membranes
SPSS: Statistical Package for the Social Sciences
